# Supplementary figures and images for: Online activity of mosques and Muslims in the Netherlands: A study of Facebook, Instagram, YouTube and Twitter
Source: PLoS One. 2021 Jul 22;16(7):e0254881. doi: 10.1371/journal.pone.0254881 (PMC8297904; doi:10.1371/journal.pone.0254881)

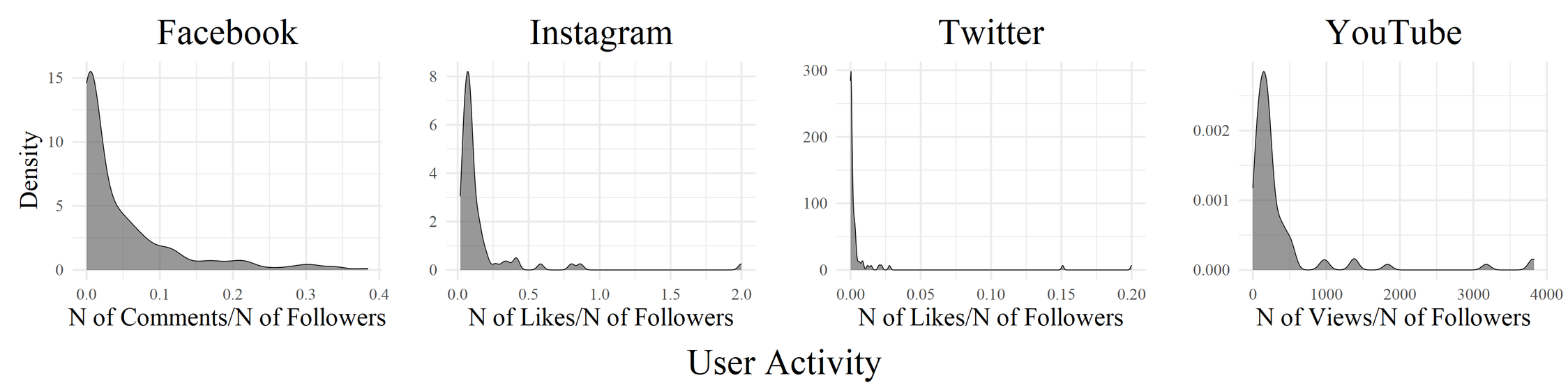

Supplement: S1 Fig — (TIF) [file pone.0254881.s001.tif]
