# Supplementary material for: Online activity of mosques and Muslims in the Netherlands: A study of Facebook, Instagram, YouTube and Twitter
Source: PLoS One. 2021 Jul 22;16(7):e0254881. doi: 10.1371/journal.pone.0254881 (PMC8297904; doi:10.1371/journal.pone.0254881)
Supplement: S1 Text — (DOCX) [file pone.0254881.s002.docx]

**S1 Text. Results procedural note.**

We present findings from regression models of online presence of mosques (S1 Table), their online activity (S2 Table), their number of followers (S3 Table), the activity of followers (S4 Table), and geographic distance of followers (S5 Table). Given the nature and distribution of these data (count, skewed), we additionally run Poisson models. Results of these analyses lead to the same conclusions, and given the ease of interpreting OLS coefficients, we present findings of the OLS models.
