# Supplementary material for: Online activity of mosques and Muslims in the Netherlands: A study of Facebook, Instagram, YouTube and Twitter
Source: PLoS One. 2021 Jul 22;16(7):e0254881. doi: 10.1371/journal.pone.0254881 (PMC8297904; doi:10.1371/journal.pone.0254881)
Supplement: S2 Text — (DOCX) [file pone.0254881.s003.docx]

**S2 Text. Data collection.**

**S2.1 Mosques**

If a mosque was present on a certain social media platform, we measured their activity. Given the different nature of the four platforms, the indicators for activity depend on the specific platform.

- *Facebook activity* refers to the average number of posts per week, based on the last 20 posts of the mosque. This was manually assessed by dividing the number 20 by the period in days between the date of the 20st most recent post and the date of data collection, and subsequently multiplying this number by 7 to get a measure of average number of posts per week*.* Example: if there are 30 days between the most recent post and the 20st most recent post, then this implies (20/30) * 7 = 4.67 posts per week.
- *Twitter activity* is a variable capturing the average number of tweets per week, based on the entire history of the mosque account. This measure was calculated by dividing the total number of tweets by the period in days between the first tweet and the date of data collection, and subsequently multiplying by 7. Activities of the mosques’ Twitter pages were gathered with Twitter API.
- *Instagram activity* is a variable indicating the measure average number of posts per week, based on the entire history of the mosque account. Like Twitter activity, this measure was calculated by dividing the total number of posts by the period in days between the first post and the date of data collection and then multiplied by 7. Data were manually collected.
- *YouTube activity* is a variable measuring the average number of videos posted per week, based on the entire history of the account. Data were collected with the YouTube Data API.

**S2.2 Followers: Numbers and Activity**
With respect to the social media platforms, we constructed popularity measures for those mosques that were present on these social media platforms.

- *Facebook popularity* indicates the number of users who liked the mosque page, collected through the Netvizz application metric [1]. Netvizz uses the Facebook API for extracting the available data from the selected Facebook pages.
- *Twitter popularity*, *Instagram popularity* and *YouTube popularity* all capture the total number of followers on the respective platforms. The number of followers on Instagram were manually gathered, while the data for Twitter and YouTube were automatically retrieved with the help of the corresponding API’s.

To examine how active the followers of the mosque pages on the various social media platforms are, we constructed normalized measures that capture this dimension.

- *Facebook user activity* is calculated by dividing the number of people who placed comments on the mosque Facebook page by the total number of likes (followers) of that page. This results in a normalized measure of how active the followers of that mosque page in fact are. The amount of people that placed comments was retrieved trough the Netvizz app.
- *Twitter user activity* is calculated by average number of likes divided by the total number of followers, using the Twitter API.
- *Instagram user activity* was collected using the average likes of the last 10 posts of a mosque. In cases of fewer placed posts or up to 12 posts, all posts were taken into account to compute the average. For private accounts it was not possible to calculate the average number of likes. Like Facebook and Twitter, a normalized measure was constructed by dividing the average likes by the total number of followers.
- *YouTube user activity* was based on the average number of views divided it by total number of subscribers (followers).

**S2.3 Twitter**

*Geolocation*

On Twitter, information on geolocation is provided as plain text on their profile page, so it may be indicated as “Amsterdam” or “Netherlands” or “Istanbul”, without indication of longitude and latitude. Sometimes users indicate location as “somewhere in the world”, “planet Earth” “rainbow” etc., which self-evidently are not useful for the analysis. Therefore, those undefinable or undetectable places were not taken into account. After removing those irrelevant locations (n = 260) and missing data, we were able to identify 48% of the users with geolocations (5474 out of 11357). As a next step, we used Google Maps API to attach coordinates to the places indicated in the profiles. Based on these user coordinates and addresses of mosques, we were able to calculate the distance from a mosque to the place of living of their followers.

To assess if Twitter users following Dutch mosques live in the Netherlands, we proceeded as follows. Each follower in our dataset was already assigned with the pair of geodata coordinates – longitude and latitude. The next step was to derive the name of the location, to which those coordinates belong. This was done with the help of the R package ‘geonames’, which allows prescribing geodata with the name of the place on the level of country, region, city or small towns. After getting the information about the country names, the ratio of the users from the Netherlands was calculated by dividing the number of followers with the location indicated inside the Netherlands, by the overall number of users with the filled-in location per each mosque.

*Community structure*

We explored interconnectedness of Dutch mosques on Twitter by constructing a bipartite network of mosques and their followers. We derived unimodal projection of the bipartite network, where the link between two pages of mosques was placed based on the followers, who follow both of those mosques; so the weight of the link increases with the number of shared followers. Overall, 75 out of 78 mosques are connected by the followers, having from 567 to 1 subscribers in common, with the exception of 3 mosques (extremely small number of followers, 2 to 5) with no links to the main component of the network. For the convenience of visual inspection, we visualized the networks via using *Cytoscape* software.

**References**

1. Rieder B. Studying Facebook via data extraction: the Netvizz application, WebSci '13 Proceedings of the 5th Annual ACM Web Science Conference (pp. 346-355). New York: ACM. . 2013: 346-355.

stylefix
