# Supplementary material for: Online activity of mosques and Muslims in the Netherlands: A study of Facebook, Instagram, YouTube and Twitter
Source: PLoS One. 2021 Jul 22;16(7):e0254881. doi: 10.1371/journal.pone.0254881 (PMC8297904; doi:10.1371/journal.pone.0254881)
Supplement: S1 Table — (DOCX) [file pone.0254881.s004.docx]

**S1 Table.** Regression analysis of online presence of mosques in the Netherlands.

|  | **Website (0 = No; 1 = Yes)** | | | | | **Facebook (0 = No; 1 = Yes)** | | | | |
| --- | --- | --- | --- | --- | --- | --- | --- | --- | --- | --- |
|  | Coeff | SE | p-value | CI (2.5%; 97.5%) | OR | Coeff | SE | p-value | CI  (2.5%; 97.5%) | OR |
| *Ethnic group* |  |  |  |  |  |  |  |  |  |  |
| Turkey (ref.) |  |  |  |  |  |  |  |  |  |  |
| Morocco | 0.355 | 0.208 | 0.089 | **-**0.053; 0.764 | 1.426 | -0.590 | 0.214 | 0.006 | -1.011;  -0.172 | 0.554 |
| Other | 0.755 | 0.267 | 0.005 | 0.237;  1.287 | 2.127 | -0.646 | 0.264 | 0.014 | -1.164; -0.128 | 0.524 |
| *Strictness* |  |  |  |  |  |  |  |  |  |  |
| Salafist | 0.738 | 0.495 | 0.136 | -0.184; 1.790 | 2.092 | 0.299 | 0.47 | 0.504 | -0.565;  1.211 | 1.349 |
| Non-Salafist (ref.) |  |  |  |  |  |  |  |  |  |  |
|  |  |  |  |  |  |  |  |  |  |  |
| Pseudo R2 | 0.020 |  |  |  |  | 0.016 |  |  |  |  |
| N | 478 |  |  |  |  | 478 |  |  |  |  |

|  | **Twitter (0 = No; 1 = Yes)** | | | | | **Instagram (0 = No; 1 = Yes)** | | | | |
| --- | --- | --- | --- | --- | --- | --- | --- | --- | --- | --- |
|  | Coeff | SE | p-value | CI (2.5%; 97.5%) | OR | Coeff | SE | p-value | CI (2.5%; 97.5%) | OR |
| *Ethnic group* |  |  |  |  |  |  |  |  |  |  |
| Turkey (ref.) |  |  |  |  |  |  |  |  |  |  |
| Morocco | 0.428 | 0.295 | 0.146 | -0.150; 1.010 | 1.535 | -1.654 | 0.349 | 0.000 | -2.387;  -1.006 | 0.191 |
| Other | 0.651 | 0.341 | 0.057 | -0.029; 1.316 | 1.918 | -0.996 | 0.374 | 0.008 | -1.782;  -0.302 | 0.369 |
| *Strictness* |  |  |  |  |  |  |  |  |  |  |
| Salafist | 1.768 | 0.455 | 0.000 | 0.882; 2.683 | 5.858 | -0.773 | 1.052 | 0.463 | -3.686; 0.880 | 0.462 |
| Non-Salafist (ref.) |  |  |  |  |  |  |  |  |  |  |
|  |  |  |  |  |  |  |  |  |  |  |
| Pseudo R2 | 0.055 |  |  |  |  | 0.079 |  |  |  |  |
| N | 478 |  |  |  |  | 478 |  |  |  |  |

Tests are two-sided. Threshold for significance = .05. Logistic regression models.

Tests are two-sided. Threshold for significance = .05. Logistic regression models.

|  | **YouTube (0 = No; 1 = Yes)** | | | | | **Average (scale 0-5)** | | | |
| --- | --- | --- | --- | --- | --- | --- | --- | --- | --- |
|  | Coeff | SE | p-value | CI (2.5%; 97.5%) | OR | Coeff | SE | p-value | CI (2.5%; 97.5%) |
| *Ethnic group* |  |  |  |  |  |  |  |  |  |
| Turkey (ref.) |  |  |  |  |  |  |  |  |  |
| Morocco | 0.981 | 0.275 | 0.000 | 0.448;  1.531 | 2.667 | -0.061 | 0.141 | 0.665 | -0.337; 0.215 |
| Other | 0.776 | 0.337 | 0.021 | 0.105;  1.434 | 2.173 | 0.071 | 0.175 | 0.686 | -0.273; 0.415 |
| *Strictness* |  |  |  |  |  |  |  |  |  |
| Salafist | -0.165 | 0.529 | 0.755 | -1.308; 0.806 | 0.848 | 0.544 | 0.297 | 0.068 | -0.041; 1.127 |
| Non-Salafist (ref.) |  |  |  |  |  |  |  |  |  |
|  |  |  |  |  |  |  |  |  |  |
| Pseudo R2 | 0.03 |  |  |  |  | 0.008 |  |  |  |
| N | 478 |  |  |  |  | 478 |  |  |  |

Tests are two-sided. Threshold for significance: .05. Logistic regression model (YouTube) and OLS regression model (average scale 0-5).
