# Supplementary material for: Online activity of mosques and Muslims in the Netherlands: A study of Facebook, Instagram, YouTube and Twitter
Source: PLoS One. 2021 Jul 22;16(7):e0254881. doi: 10.1371/journal.pone.0254881 (PMC8297904; doi:10.1371/journal.pone.0254881)
Supplement: S2 Table — (DOCX) [file pone.0254881.s005.docx]

**S2 Table**. Regression of activity of mosques on social media platforms in the Netherlands, by ethnic-national orientation and strictness.

| **Facebook (average posts per week)** | | | | |
| --- | --- | --- | --- | --- |
|  | Coeff | SE | p-value | CI (2.5%; 97.5%) |
| Constant | 3.267 | 0.263 | 0.000 | 2.749; 3.785 |
| *Ethnic group* |  |  |  |  |
| Turkey (ref.) |  |  |  |  |
| Morocco | -0.963 | 0.440 | 0.029 | -1.828; -0.097 |
| Other | -0.959 | 0.554 | 0.084 | -2.049; 0.131 |
| *Strictness* |  |  |  |  |
| Salafist | 0.036 | 0.909 | 0.969 | -1.755; 1.826 |
| Non-Salafist (ref.) |  |  |  |  |
|  |  |  |  |  |
| R2 | 0.011 |  |  |  |
| N | 282 |  |  |  |

Tests are two-sided. Threshold for significance = .05. OLS regression models.

| **Twitter (average tweets per week)** | | | | |
| --- | --- | --- | --- | --- |
|  | Coeff | SE | p-value | CI (2.5%; 97.5%) |
| Constant | 1.534 | 0.655 | 0.022 | 0.228; 2.841 |
| *Ethnic group* |  |  |  |  |
| Turkey (ref.) |  |  |  |  |
| Morocco | -0.666 | 0.876 | 0.450 | -2.414; 1.081 |
| Other | 1.805 | 1.009 | 0.078 | -0.208;  3.818 |
| *Strictness* |  |  |  |  |
| Salafist | 1.909 | 1.048 | 0.073 | -0.182; 4.001 |
| Non-Salafist (ref.) |  |  |  |  |
|  |  |  |  |  |
| R2 | 0.095 |  |  |  |
| N | 73 |  |  |  |

Tests are two-sided. Threshold for significance = .05. OLS regression models.

| **Instagram (average posts per week)** | | | | |
| --- | --- | --- | --- | --- |
|  | Coeff | SE | p-value | CI (2.5%; 97.5%) |
| Constant | 1.283 | 0.203 | 0.000 | 0.876; 1.689 |
| *Ethnic group* |  |  |  |  |
| Turkey (ref.) |  |  |  |  |
| Morocco | -0.995 | 0.538 | 0.069 | -2.070; 0.079 |
| Other | -0.108 | 0.567 | 0.849 | -1.239;  1.022 |
| *Strictness* |  |  |  |  |
| Salafist | 0.035 | 1.576 | 0.982 | -3.110;  3.181 |
| Non-Salafist (ref.) |  |  |  |  |
|  |  |  |  |  |
| R2 | 0.010 |  |  |  |
| N | 72 |  |  |  |

Tests are two-sided. Threshold for significance = .05. OLS regression models.

| **YouTube (average videos posted per week)** | | | | |
| --- | --- | --- | --- | --- |
|  | Coeff | SE | p-value | CI (2.5%; 97.5%) |
| Constant | 0.082 | 0.031 | 0.009 | 0.021; 0.143 |
| *Ethnic group* |  |  |  |  |
| Turkey (ref.) |  |  |  |  |
| Morocco | 0.063 | 0.039 | 0.114 | -0.015; 0.142 |
| Other | 0.061 | 0.047 | 0.200 | -0.033 0.154 |
| *Strictness* |  |  |  |  |
| Salafist | 0.171 | 0.073 | 0.022 | 0.025; 0.316 |
| Non-Salafist (ref.) |  |  |  |  |
|  |  |  |  |  |
| R2 | 0.075 |  |  |  |
| N | 88 |  |  |  |

Tests are two-sided. Threshold for significance = .05. OLS regression models.
