# Supplementary material for: Online activity of mosques and Muslims in the Netherlands: A study of Facebook, Instagram, YouTube and Twitter
Source: PLoS One. 2021 Jul 22;16(7):e0254881. doi: 10.1371/journal.pone.0254881 (PMC8297904; doi:10.1371/journal.pone.0254881)
Supplement: S3 Table — (DOCX) [file pone.0254881.s006.docx]

**S3 Table**. Regression of number of followers on social media platforms in the Netherlands, by ethnic group and strictness.

| **Facebook** | | | | |
| --- | --- | --- | --- | --- |
|  | Coeff | SE | p-value | CI (2.5%; 97.5%) |
| Constant | 946.233 | 128.295 | 0.000 | 693.585; 1198.880 |
| *Ethnic group* |  |  |  |  |
| Turkey (ref.) |  |  |  |  |
| Morocco | 361.517 | 209.272 | 0.085 | -50.597; 773.632 |
| Other | 661.500 | 252.794 | 0.009 | 163.680; 1159.320 |
| *Strictness* |  |  |  |  |
| Salafist | 1041.827 | 412.545 | 0.012 | 229.413; 1854.240 |
| Non-Salafist (ref.) |  |  |  |  |
|  |  |  |  |  |
| R2 | 0.053 |  |  |  |
| N | 260 |  |  |  |

Tests are two-sided. Threshold for significance = .05. OLS regression model.

| **Twitter** | | | | |
| --- | --- | --- | --- | --- |
|  | Coeff | SE | p-value | CI (2.5%; 97.5%) |
| Constant | 70.654 | 150.662 | 0.640 | -229.353; 370.660 |
| *Ethnic group* |  |  |  |  |
| Turkey (ref.) |  |  |  |  |
| Morocco | 90.927 | 208.203 | 0.664 | -323.658; 505.513 |
| Other | 566.839 | 232.751 | 0.017 | 103.374; 1030.305 |
| *Strictness* |  |  |  |  |
| Salafist | 123.530 | 243.825 | 0.614 | -361.987;  609.047 |
| Non-Salafist (ref.) |  |  |  |  |
|  |  |  |  |  |
| R2 | 0.059 |  |  |  |
| N | 81 |  |  |  |

Tests are two-sided. Threshold for significance = .05. OLS regression model.

| **Instagram** | | | | |
| --- | --- | --- | --- | --- |
|  | Coeff | SE | p-value | CI (2.5%; 97.5%) |
| Constant | 398.098 | 51.265 | 0.000 | 296.037; 500.160 |
| *Ethnic group* |  |  |  |  |
| Turkey (ref.) |  |  |  |  |
| Morocco | -226.098 | 136.601 | 0.102 | -498.050; 45.853 |
| Other | -39.098 | 136.601 | 0.775 | -311.050;  232.853 |
| *Strictness* |  |  |  |  |
| Salafist | -114.000 | 419.938 | 0.787 | -950.033; 722.033 |
| Non-Salafist (ref.) |  |  |  |  |
|  |  |  |  |  |
| R2 | 0.004 |  |  |  |
| N | 82 |  |  |  |

Tests are two-sided. Threshold for significance = .05. OLS regression model.

| **YouTube** | | | | |
| --- | --- | --- | --- | --- |
|  | Coeff | SE | p-value | CI (2.5%; 97.5%) |
| Constant | 17.962 | 141.568 | 0.899 | -263.562; 299.486 |
| *Ethnic group* |  |  |  |  |
| Turkey (ref.) |  |  |  |  |
| Morocco | 324.577 | 182.764 | 0.079 | -38.869; 688.023 |
| Other | 400.372 | 221.338 | 0.074 | -39.783;  840.527 |
| *Strictness* |  |  |  |  |
| Salafist | -39.938 | 342.895 | 0.908 | -721.823;  641.946 |
| Non-Salafist (ref.) |  |  |  |  |
|  |  |  |  |  |
| R2 | 0.015 |  |  |  |
| N | 88 |  |  |  |

Tests are two-sided. Threshold for significance = .05. OLS regression model.
