# Supplementary material for: Online activity of mosques and Muslims in the Netherlands: A study of Facebook, Instagram, YouTube and Twitter
Source: PLoS One. 2021 Jul 22;16(7):e0254881. doi: 10.1371/journal.pone.0254881 (PMC8297904; doi:10.1371/journal.pone.0254881)
Supplement: S4 Table — (DOCX) [file pone.0254881.s007.docx]

**S4 Table**. Regression of user activity of mosques on social media platforms in the Netherlands, by ethnic group and strictness.

| **Facebook**  **(N of comments/N of followers)** | | | | |
| --- | --- | --- | --- | --- |
|  | Coeff | SE | p-value | CI (2.5%; 97.5%) |
| Constant | 0.063 | 0.007 | 0.000 | 0.050; 0.076 |
| *Ethnic group* |  |  |  |  |
| Turkey (ref.) |  |  |  |  |
| Morocco | -0.020 | 0.011 | 0.069 | -0.041;  0.002 |
| Other | -0.045 | 0.014 | 0.001 | -0.072;  -0.017 |
| *Strictness* |  |  |  |  |
| Salafist | -0.018 | 0.023 | 0.441 | -0.063;  0.028 |
| Non-Salafist (ref.) |  |  |  |  |
|  |  |  |  |  |
| R2 | 0.041 |  |  |  |
| N | 235 |  |  |  |

Tests are two-sided. Threshold for significance = .05. OLS regression model.

| **Twitter**  **(N of likes/N of followers)** | | | | |
| --- | --- | --- | --- | --- |
|  | Coeff | SE | p-value | CI (2.5%; 97.5%) |
| Constant | 0.002 | 0.006 | 0.775 | -0.011;  0.015 |
| *Ethnic group* |  |  |  |  |
| Turkey (ref.) |  |  |  |  |
| Morocco | 0.008 | 0.009 | 0.339 | -0.009;  0.025 |
| Other | 0.014 | 0.010 | 0.167 | -0.006;  0.0332 |
| *Strictness* |  |  |  |  |
| Salafist | -0.010 | 0.010 | 0.312 | -0.030;  0.010 |
| Non-Salafist (ref.) |  |  |  |  |
|  |  |  |  |  |
| R2 | 0.006 |  |  |  |
| N | 72 |  |  |  |

Tests are two-sided. Threshold for significance = .05. OLS regression model.

| **Instagram**  **(N of likes/N of followers)** | | | | |
| --- | --- | --- | --- | --- |
|  | Coeff | SE | p-value | CI (2.5%; 97.5%) |
| Constant | 0.091 | 0.033 | 0.008 | 0.024; 0.157 |
| *Ethnic group* |  |  |  |  |
| Turkey (ref.) |  |  |  |  |
| Morocco | 0.333 | 0.088 | 0.000 | 0.157; 0.509 |
| Other | 0.224 | 0.093 | 0.019 | 0.039; 0.408 |
| *Strictness* |  |  |  |  |
| Salafist | -0.379 | 0.258 | 0.146 | -0.893;  0.135 |
| Non-Salafist (ref.) |  |  |  |  |
|  |  |  |  |  |
| R2 | 0.176 |  |  |  |
| N | 72 |  |  |  |

Tests are two-sided. Threshold for significance = .05. OLS regression model.

| **YouTube (N of views/N of followers)** | | | | |
| --- | --- | --- | --- | --- |
|  | Coeff | SE | p-value | CI (2.5%; 97.5%) |
| Constant | 856.457 | 135.451 | 0.000 | 586.796; 1126.118 |
| *Ethnic group* |  |  |  |  |
| Turkey (ref.) |  |  |  |  |
| Morocco | -668.313 | 173.403 | 0.000 | -1013.531;  -323.095 |
| Other | -608.083 | 204.426 | 0.004 | -1015.064;  -201.102 |
| *Strictness* |  |  |  |  |
| Salafist | -57.895 | 310.028 | 0.852 | -675.113;  559.322 |
| Non-Salafist (ref.) |  |  |  |  |
|  |  |  |  |  |
| R2 | 0.147 |  |  |  |
| N | 82 |  |  |  |

Tests are two-sided. Threshold for significance = .05. OLS regression model.
