# Supplementary material for: Online activity of mosques and Muslims in the Netherlands: A study of Facebook, Instagram, YouTube and Twitter
Source: PLoS One. 2021 Jul 22;16(7):e0254881. doi: 10.1371/journal.pone.0254881 (PMC8297904; doi:10.1371/journal.pone.0254881)
Supplement: S5 Table — (DOCX) [file pone.0254881.s008.docx]

**S5 Table.** Regression analysis of geographic distance between the mosque and the place of living of their followers on Twitter.

|  | Coeff | SE | p-value | CI (2.5%; 97.5%) |
| --- | --- | --- | --- | --- |
| Constant | 525.808 | 86.255 | 0.000 | 356.723;  694.894 |
| *Ethnic group* |  |  |  |  |
| Turkey (ref.) |  |  |  |  |
| Morocco | 1090.085 | 106.274 | 0.000 | 881.757; 1298.413 |
| Other | 3324.281 | 97.796 | 0.000 | 3132.572; 3515.990 |
| *Strictness* |  |  |  |  |
| Salafist | -552.763 | 73.357 | 0.000 | -696.564;  -408.962 |
| Non-Salafist (ref.) |  |  |  |  |
|  |  |  |  |  |
| R2 | 0.205 |  |  |  |
| N | 7329 |  |  |  |

Tests are two-sided. Threshold for significance = .05. OLS regression model.
