# Supplementary material for: Online activity of mosques and Muslims in the Netherlands: A study of Facebook, Instagram, YouTube and Twitter
Source: PLoS One. 2021 Jul 22;16(7):e0254881. doi: 10.1371/journal.pone.0254881 (PMC8297904; doi:10.1371/journal.pone.0254881)
Supplement: Attachment — Submitted filename: Response to Reviewers.docx [file pone.0254881.s009.docx]

**Response to Reviewers**

We would like to thank the reviewers for taking the time to read our paper and to give feedback. It really helped us to improve the paper.

Reviewer #1: 

1. Average distance is currently calculated country-wide but perhaps can be refined further into regions or cities etc to identify which mosques have followers from other cities indicating hubs.

This is an interesting idea. In the paper, we look at the geographic distance between the mosques in the Netherlands and the place of living of their ‘followers’ on Twitter. We provide different pieces of information. First, we show how many of the followers of the mosques actually live in the Netherlands or abroad. This gives us a first idea of how nationally or internationally embedded followers of mosques are that are located in the Netherlands. Then, we take a closer look at those mosque-followers who live in the Netherlands, providing two further pieces of information: (a) what is their average distance to the mosque, (b) how many live very nearby (less than 5 kilometer from the mosque). We like the suggestion to go beyond this analysis and also look at the cities from which mosques attract followers. Unfortunately, this raises an issue of ethics and privacy, because revealing the geographical location of mosques would not be in line with GDPR rules. The data collection for the project has been approved by the ethics committee of the university, but this approval does not include revealing geographical data which could lead to identification of mosques. Therefore, we cannot provide information of the kind that “the mosque located in city X appear to be a major hub in the Netherlands,” or “city X attracts followers from Y”, because if there is only one such mosque in city X, and that happens, we effectively reveal the identity of the mosque. That’s why we present results on the share of mosque-followers living nearby.

2. Have the ownership of social media pages certified? It may be the case that a Facebook page is not owned by the mosque.

This is a good point. We checked the social media pages extensively, one by one, and included pages on mosques in the Netherlands that are created by (a) an organizational account of the mosque, or (b) a person on behalf of the mosque. To qualify for (b), (a) should not be present on the same social media platform, and, moreover, it should be clear from the content and number of followers that it is in fact the main social media account (e.g., on Facebook) that is used to spread information about the mosque. We clarify this in the Revision.

3. Authors indicate that YouTube is not used frequently by the mosques but Facebook is. Perhaps further analysis can be conducted in terms of type of content? So it will be interesting to see the type of content published on Facebook and other forums.

This is an interesting suggestion. We looked at many Facebook pages of mosques, and what we discovered is that actually a lot of visual, non-textual information was provided. Pictures and videos of meetings, lectures, gatherings and social events. The content of these visual data complicates the analysis, because the amount of visual and textual data strongly varies from mosque to mosque (e.g., one mosque posting mainly pictures and videos, while another mosque posts text). Such a study, however interesting, goes beyond the scope of this paper. It is for these reasons that we have refrained from such an analysis in this paper, but we do mention this idea as an interesting step forward for follow-up research.

4. Some figures require improvement with respect to their quality.

We checked all figures, and discovered one figure (Fig 3) which was of lower quality (144dpi instead of 300dpi). We corrected this.

Reviewer #2:

1. A review and comparison of the related works are missing. What is the gap in the current literature? What is the scientific value and novelty of the proposed solution? What is the relevance of this paper to the journal?

We agree that the contributions of this study could be better articulated. We have completely rewritten the Introduction, included a thorough review of previous work, and clearly outlined how this paper significantly contributes to the state-of-the art.

2. What is the analysis of online activities of different minority communities on social media? How to avoid any bias in such an analysis? How the results from such analysis can be used to ensure better facilities, social justice, equality. How to avoid any ethical issues, or avoid bias, stigmatization on minorities [1]. [1] S. Loue, "Ethical Issues in Conducting Research on Religion and Spirituality",

We read the paper suggested by the reviewer, and what it says is in line with GDPR guidelines. To clarify, our paper has been reviewed and approved by the University Ethics Committee, which means that our work presented here is 100% following GDPR rules.

3. How can such analysis be used to measure equality, diversity, social justice for minorities? What is the purpose of this analysis? What methods and algorithms can be used to carry out such analyses? There are a lot of open-ended questions that may arise which should be answered. This paper should be thoroughly revised and should be re-submitted.

We’re not sure if we understand the points the reviewer wants to make. The aims of the paper are not to measure equality, diversity or social justice for minorities. We clearly formulate the aims and background of the study, research questions, contributions, theory and hypotheses, methods, results and implications. We also submitted Supplemental materials, which contain more detailed information on the methods and algorithms that we used.

Reviewer #3: 

1. First, the piece is very descriptive and lacks a positivist research focus. This is quite problematic for an article of this nature. Oftentimes descriptive pieces are either uncovering empirically novel insights or data collection strategies that are original. Unfortunately, neither applies here.

We strongly embrace a theory-driven, analytical (‘positivist’) research approach. The questions we address in this study are not only descriptive. On the contrary, we also raise explanatory questions, we discuss several theoretical mechanisms that help to understand differences between Muslim groups in their adoption of online platforms and derive several hypotheses which are then tested in the quantitative analyses. We suspect that we have not emphasized this theoretical part very well in the previous version of this paper, so it may have indeed appeared as if we only introduced descriptive material. In this Revision, we have emphasized more strongly the theoretical contributions of the paper and included an entire section on Theory and Hypotheses. Note that with respect to the descriptive analyses, we’re the first to present findings from a large-scale comparative study on Muslims adoption of online platforms. This is a significant contribution to most work in this field, which takes a more qualitative, case-study approach (from which it’s hard to make inferences to other groups and platforms).

2. Second, the lack of theoretical engagement is problematic, because it is unclear what to take away from the different research questions. Why should we care about the differences between Turkish and Moroccan mosques? What do theories about immigrant assimilation/acculturation suggest? Or is this about Islamic brand/theology?

We agree that in the previous version, these theoretical questions were not articulated very well. We also agree that the discussion of previous work and theories could be more elaborate. We have therefore entirely rewritten the Introduction, incorporating a much strong review of previous work. We have also clearly formulated the three contributions of this paper and included a separate section on Theory and Hypotheses.

3. Third, why should we be surprised that Salafist mosques have a significant online presence? Most right-wing/fundamental/radical groups tend to exhibit similar trends.

In this paper, we look not only at the online presence and activity of mosques, but also at their online ‘followers’. A key question regarding the status of Muslim minority groups in Western Europe, is how they respond to the secular and assimilationist forces. What we find is that not only are the orthodox Salafist mosques more active online, but also that they have more online followers, and that these followers make up a more cohesive group: they cluster together in the same areas, and online they make up a cohesive web of in-group ties. This study is the first to show that the most-orthodox Muslim groups have been more successful than other Muslim groups in building an online organizational structure that responds to the secularizing-assimilationist forces in the Netherlands.

4. Finally, I am not entirely convinced that mosque “participants” are captured adequately. The ms takes twitter followers as a measure of participation. This is a major unsubstantiated assumption. Many follow mosques, even though they aren’t participants. So I wonder about the validity of this measure. There should have been an effort to substantiate this claim.

This is a very good point. We agree that a ‘follower’ of a mosque online does not imply that s/he also participates in meetings of that mosque offline. Everyone can follow mosques online, and thus a ‘follower’ of a Turkish mosque on Twitter does not mean that this follower attends meetings of that mosque. We clarify this issue in the paper, note that this possible ‘bias’ applies to all mosques we study (we have no evidence to suggest it affects some mosques more than other mosques), and ceteris paribus test the hypotheses.
