# Supplementary material for: Online activity of mosques and Muslims in the Netherlands: A study of Facebook, Instagram, YouTube and Twitter
Source: PLoS One. 2021 Jul 22;16(7):e0254881. doi: 10.1371/journal.pone.0254881 (PMC8297904; doi:10.1371/journal.pone.0254881)
Supplement: Attachment — Submitted filename: Response to Reviewers.docx [file pone.0254881.s010.docx]

**Response to Reviewers**

We would like to thank Reviewer #3 once more for taking the time to read our paper and to give feedback.

1. One suggestion is to further build the theory being tested. It appears that the Turkish mosques have better resources than the Moroccan ones as the author stipulates. One way to motivate the study is to ask whether organizational resources or ideology serve as better predictors of online networks. This would be directly engaging theories of resource mobilization and the role of affluence in acculturation/assimilation models against ideological commitments which may structure ingroup linkages/biases.

This is a good suggestion. We followed your advice and included already in the introduction this theoretical contrast between organizational resources and ideology as drivers for online activity.

2. I believe that Moroccans are a more recent community (?) So that can be juxtaposed against the Turks who have probably been in the country for a longer period of time. If this is correct, and there are no differences between the two communities, it illustrates the time in host country is less consequential to online networking.

Turks and Moroccans arrived at the same time, as part of the ‘guest-worker’ program of the Dutch government in the 1960s. This is now clarified in the introduction.

3. To examine the resource-based argument, the author can proceed to compare Moroccan to Turkish mosques. To examine the ideological explanation, the author will look at Salafist/non-Salafist mosques as is already done in the paper.

This is what we now do in the paper, see Hypothesis 1 (resource argument) and Hypothesis 2 and 3 (ideology).

4. (Please note that Salafists only constitute 5% of mosques, so this should be highlighted. Further, the author should provide the coding scheme for Salafist vs non-Salafist mosques.)

We now highlight the 5% in the revised manuscript. Regarding coding of Salafist/non-Salafist mosques, we rely on previous work and include references to these studies in the current paper. We didn’t do the coding ourselves, therefore, but instead relied on previous work that coded mosques as Salafist based on a variety of sources, such as Salafist self-identification of mosques, anthropological fieldwork, and information on the ideology of mosques. We were able to match the names of these mosques mentioned in these studies to our list of 478 mosques.

5. The author can also look at the role of time in host country, as yet a third dimension of theoretical interest.

This would be interesting to study, but because the groups with enough mosques for analysis (Turks, Moroccans) arrived at the same time, we cannot study it.

6. Other Points smaller: On page 11, there’s a discussion of Pakistani mosques, but it’s unclear whether these are more comparable to Turkish or Moroccan mosques?

That’s a difficult question, we know very little about Pakistani, their organizational structure and ideology -it’s a very, very small group in the Netherlands (< 25,000 immigrants and children of immigrants are from Pakistani origin). It’s remarkable that they are so-well organized online, that’s why we mentioned it in the descriptive part of the paper.

7. Page 18: 5-kilometer distance to mosque (%)-----it appears a larger percentage of non-Salafists live in this radius. So I would relax the language here about Salafists living closer to mosques.

We have changed the text accordingly.
